# The Influence of MTHFR Polymorphism on Gray Matter Volume in Patients With Amnestic Mild Cognitive Impairment

**DOI:** 10.3389/fnins.2021.778123

**Published:** 2021-11-30

**Authors:** Mengzhe You, Xia Zhou, Wenwen Yin, Ke Wan, Wei Zhang, Chenchen Li, Mingxu Li, Wenhao Zhu, Xiaoqun Zhu, Zhongwu Sun

**Affiliations:** Department of Neurology, First Affiliated Hospital of Anhui Medical University, Hefei, China

**Keywords:** methylenetetrahydrofolate reductase, Alzheimer’s disease, amnestic mild cognitive impairment, apolipoprotein E, voxel-based morphometry

## Abstract

The methylenetetrahydrofolate reductase (MTHFR) gene has been associated with Alzheimer’s disease (AD) pathogenesis. Amnestic mild cognitive impairment (aMCI) represents a prodromal stage of dementia and involves a high risk of progression into AD. Although the effects of the apolipoprotein E (APOE) gene on structural alterations in aMCI have been widely investigated, the effects of MTHFR C677T and interaction effects of MTHFR × APOE genotypes on gray matter atrophy in aMCI remain largely unknown. In the present study, 60 aMCI patients and 30 healthy controls were enrolled, and voxel-based morphometry analysis was performed to inspect the effects of diagnosis, different genotypes, and their interactions on gray matter atrophy. The results showed that aMCI patients had significant gray matter atrophy involving the bilateral hippocampus, the right parahippocampal gyrus, and the left superior temporal gyrus compared with healthy controls. Besides, a substantial reduction in gray matter volume was observed in the right hippocampus region in APOE ε4 carriers from the aMCI group, compared with APOE ε4 non-carriers. A significant interaction was found between diagnosis and MTHFR C677T genotype on the right precuneus in healthy controls and aMCI patients not carrying APOE ε4 allele. Our findings may provide new evidence substantiating the genetic effects of MTHFR C677T on brain structural alternation in patients with aMCI.

## Introduction

Alzheimer’s disease (AD) is the most common neurodegenerative disorder globally, accounting for 60 to 80% of dementia cases. Individuals who suffer from AD experience progressive memory decline and impairment of other cognitive functions (Scheltens et al., 2021). Amnestic mild cognitive impairment (aMCI) is a transitional state between normal age-related cognitive decline and dementia, being the prodromal stage of AD (Petersen et al., 1999), and entails a high risk of progression into AD, with an estimated rate of 10 to 15% per year, compared with a rate of 1 to 2% per year for healthy older people (Petersen et al., 1999).

Apolipoprotein E (APOE) ε4 allele is an important genetic factor that increases susceptibility to sporadic AD (Scheltens et al., 2021) and has been associated with amyloid deposition, tau hyperphosphorylation, cholesterol transport (Scheltens et al., 2021), which can reportedly accelerate the progression to AD. However, the APOE ε4 allele can only account for the heritability of AD to a certain extent (Ridge et al., 2013; Bellenguez et al., 2017). More than 40 AD-associated risk alleles have been found in genome-wide association studies (Jansen et al., 2019; Sims et al., 2020).

The methylenetetrahydrofolate reductase (MTHFR) gene is the major genetic modifier that affects the folate cycle and homocysteine metabolism. MTHFR C677T (rs1801133) is one of the most common polymorphisms of the MTHFR gene and is thought to be involved in the pathogenesis of AD (Hu et al., 2016). The C677T polymorphism, which refers to the substitution of cytosine (C) at base 677 of the MTHFR-encoding gene with thymine (T) and alanine at codon 222 replaced by valine, has been reported to result in decreased MTHFR enzyme activity (Frosst et al., 1995), leading to elevated homocysteine concentrations. Hyperhomocysteinemia is widely acknowledged to be a risk factor of AD (Smith et al., 2018), which influences DNA methylation (Coppede, 2010), DNA repair (Bednarska-Makaruk et al., 2016), oxidative stress (Deep et al., 2019), amyloid β aggregation (Chung et al., 2016; Hoffman et al., 2018), tau phosphorylation (Sontag et al., 2014), vascular endothelial dysfunction (Esse et al., 2019; Wu et al., 2019), and the secretion of inflammatory mediators, especially tumor necrosis factor α, nuclear factor κB, interleukin 6 (IL-6), and IL-8 (Di Meco et al., 2019). Moreover, epigenetic level refers to the mutual gene interaction between the MTHFR gene and other genes, which may play a pivotal role in the development of AD.

Many studies have investigated the influence of single-nucleotide polymorphism of APOE on the heterogeneity in cognitive and neuroimaging findings. However, few neuroimaging studies have focused on how MTHFR polymorphism could influence brain abnormalities in aMCI patients. Interestingly, it has been reported that MTHFR C677T variant might be associated with regional brain volume reduction in white matter (WM) brain areas in MCI patients (Rajagopalan et al., 2012). In addition, the risk T allele of the MTHFR C677T was associated with a significantly increased annual rate of progressive right periventricular parietal WM atrophy, up to 1.5% per year per “T” allele. In contrast, the effect of MTHFR C677T polymorphism on gray matter (GM) atrophy in aMCI subjects remains largely unknown.

Furthermore, numerous studies have described gene–gene and gene–environment interactions in AD in recent years. Most researchers advocate that interactions between genetic factors promote progression to AD and associated brain abnormalities; however, much heterogeneity was found in the results obtained. For instance, in a study by Peng et al. (2015), where subgroup analysis was based on APOE ε4 status, MTHFR C677T polymorphism was found to be only associated with the risk of AD in APOE ε4 carriers, but not in APOE ε4 non-carriers, indicating a synergic effect between MTHFR C677T polymorphism and the APOE ε4 allele. In contrast, Stoccoro et al. (2017) documented that the MTHFR TT genotype increased the likelihood of AD in both APOE ε4 carriers and non-carriers. Nonetheless, these studies investigated only the interactions between genes at the clinical level, whereas the effects of MTHFR C677T and MTHFR × APOE genotypes interactions on GM atrophy in aMCI were largely unexplored. Given that both APOE and MTHFR genes share similar pathogenic mechanisms in AD, we sought to explore the effect of the interaction between APOE and MTHFR genes on the brain structure of aMCI patients, in addition to the influence of MTHFR C677T itself on the GM structure.

Accordingly, the main purpose of our research was to explore the effect of MTHFR C677T and the influence of the interaction between MTHFR and APOE genotypes on GM atrophy in aMCI patients. We sought to deepen our understanding of the role of susceptible genes such as MTHFR and APOE and the outcome of their interactions on GM atrophy in aMCI disease, which could importantly help to develop new strategies for disease prevention and early therapy.

## Materials and Methods

### Participants

Sixty-eight aMCI patients were recruited in the present study. The clinical diagnosis of aMCI was based on the National Institute on Aging and Alzheimer’s Association criteria (Albert et al., 2011), which included (1) subjective cognitive impairment reported by participants or their caregivers; (2) objective cognitive impairment, but no dementia; (3) Clinical Dementia Rating score of 0.5; (4) a memory function score of 1.0 to 1.5 standard deviations below the mean for their age- and education-matched peers on culturally appropriate normative data. The exclusion criteria included patients with a history of stroke, severe depression, or other neurological or psychiatric illness; WM hyperintensity with Fazekas grade ≥ 2 or a modified Hachinski ischemic score > 4; previous treatment with cholinesterase inhibitors, folate, and vitamin B to improve cognition; severe visual or auditory abnormalities; and patients with magnetic resonance contraindications.

Thirty-two healthy controls (HCs) were recruited during the same period. The healthy subjects had no underlying neurological or psychiatric disorders, no subjective or objective cognitive deficits, and no psychoactive medication use. All participants in our study were Chinese Han and right-handed. Ten participants were excluded for the following reasons: failed genotyping (*n* = 3), severe WM hyperintensity (*n* = 5), and poor quality of magnetic resonance imaging (MRI; *n* = 2). Finally, 60 aMCI patients (67.32 ± 9.44 years old; 27 males) and 30 HCs (64.76 ± 8.01 years old; 13 males) matched by gender, age, and education were eventually enrolled in the study. Our research was approved by the Institutional Ethics Committees of the First Affiliated Hospital of Anhui Medical University, and written informed consent was signed by each participant.

### Genotyping

Two milliliters of fasting blood was drawn from all participants in EDTA tubes and stored at −80°C. These blood samples were sent to Anhui Jinzhun Gene Biotechnology company for APOE gene and MTHFR C677T sequencing and typing. Genomic DNA was extracted from blood samples using Magen HiPure Blood DNA Mini Kit (D3111-03). Genotyping of rs1801133 (MTHFR), rs429358, and rs7412 (APOE) in each subject was performed using the penta-primer amplification refractory mutation system method. Primers for MTHFR and APOE genotype amplification are shown in Supplementary Table 1. After polymerase chain reaction, the plates were read by a TECAN M1000 infinite reader, and DNA sequences were analyzed using the online software snp decoder^1^. The χ^2^ test was used to assess whether the allele frequency was consistent with expectation in Hardy–Weinberg equilibrium. The statistical significance level was set at *p* < 0.05.

Because of the limited number of MTHFR TT and APOE ε4ε4 genotypes in our sample, we adopted the most appropriated genetic model (dominant model), as previously described (Chang et al., 2017, 2019). MTHFR rs1801133 polymorphism in the aMCI group was distributed between T allele carriers (*n* = 40 [66.7%], TT = 12, CT = 28), and homozygous wild-type (CC) carriers (*n* = 20 [33.3%], CC = 20). MTHFR rs1801133 polymorphism in the HC group consisted of T allele carriers (*n* = 19 [63.3%], TT = 4, CT = 15), and homozygous wild-type (CC) carriers (*n* = 11 [36.7%], CC = 11). Similarly, the distribution of APOE rs429358, rs7412 variants in the aMCI group was distributed between APOE ε4 carriers (*n* = 22 [36.7%], ε3ε4 = 16, ε2ε4 = 4, ε4ε4 = 2), and APOE ε4 non-carriers (*n* = 38 [63.3%], ε2ε2 = 0 ε2ε3 = 4, ε3ε3 = 34). In the HC group, there were 8 APOE ε4 carriers (*n* = 8 [26.7%], ε3ε4 = 8, ε2ε4 = 0, ε4ε4 = 0), and 22 APOE ε4 non-carriers (*n* = 22 [73.3%], ε2ε2 = 0, ε2ε3 = 3, ε3ε3 = 19). Allelic frequencies were arrived at Hardy–Weinberg equilibrium.

### Clinical, Biochemical, and Neuropsychological Assessments

The general condition and medical history of each participant were recorded. Three milliliters of fasting blood was collected from all participants in collection tubes for biochemical assays. The serum samples were extracted after centrifugation, and then serum homocysteine was detected by chemical luminescent immunoassay, and serum folate and vitamin B_12_ were detected by electrochemiluminescence immunoassay. All subjects were interviewed with the following questionnaires: the Mini-Mental State Examination and Montreal Cognitive Assessment were used to assess general cognitive functions; the Cambridge Cognitive Examination—Chinese version was used to assess episodic memory, attention, executive function, and visuospatial skills; the Clinical Dementia Rating was used to assess the severity of the disease; the Geriatric Depression Scale was used to assess emotion; and the Activities of Daily Living scale was used to evaluate independent living skills.

### Neuroimaging Acquisition

A 3.0-T MRI scanner (Signa HDxt, GE, Milwaukee, WI, United States) equipped with an 8-channel head coil was used to acquire three-dimensional (3D) T1-weighted structural images. Foam paddings were used to minimize head motion and earplugs to reduce scanner noise. Three-dimensional high spatial resolution T1-weighted images were obtained using the following parameters: repetition time (TR) = 9.5 ms; echo time (TE) = 3.9 ms; flip angle (FA) = 20°; 176 slices; slice thickness = 1 mm; matrix size = 512 × 512; and field of view (FOV) = 256 × 256 mm^2^. T2-weighted and T2 fluid-attenuated inversion recovery (FLAIR) sequences were performed simultaneously for each subject, the sequences were as follows: T2-weighted images (TR = 3,500 ms; TE = 85 ms; echo train length = 15; FA = 90°; axial slice thickness = 5 mm; matrix size = 512 × 512; and FOV = 230 × 184 mm^2^); T2-FLAIR images (TR = 11 s; TE = 120 ms; FA = 90°; axial slice thickness = 5 mm; matrix size = 512 × 512; and FOV = 230 × 230 mm^2^).

### MRI Data Processing and Analysis

All images were visually inspected for artifacts, motion problems, or structural abnormalities. We carried out voxel-based morphometry (VBM) analysis using the VBM8 toolbox^2^ based on SPM8 (Statistical Parametric Mapping, Wellcome Department of Imaging Neuroscience, London, United Kingdom; available online at http://www.fil.ion.ucl.ac.uk/spm), according to the following procedure. First, the 3D T1 images of the whole brain were segmented into GM, WM, and cerebrospinal fluid (CSF). Then, the segments were iteratively registered using Diffeomorphic Anatomical Registration Through Exponentiated Lie algebra (DARTEL) toolbox, and the GM images were normalized and modulated into the standard Montreal Neurological Institute space. Finally, the modulated spatial normalized GM images were smoothed with an 8-mm full-width at half maximum kernel. The total intracranial volume (TIV) was calculated by summing up the total GM, WM, and CSF volume. REST^3^ software was used to extract the GM volume of specific brain regions.

### Statistical Analysis

SPSS 22.0 software (IBM SPSS Inc., Chicago, IL, United States) was used to analyze the demographic, behavioral, and genotype data. Comparison between groups was performed by χ^2^ test for categorical variables, and two-sample *t* test and Mann-Whitney *U* test for continuous variables. Normally distributed data were expressed as mean ± standard deviation, whereas non-parametric data were represented as median and interquartile range [M (QU – QL)]. A two-tailed *p* < 0.05 was statistically significant.

A full factorial analysis of covariance was applied to analyze the effect of interactions on GM volume. First, it was performed in all study participants to explore the main effects of the diagnosis (aMCI vs. HC), MTHFR C677T genotype (CC vs. CT-TT), APOE ε4 carrier status (ε4 + vs. ε4−), and interactions between these factors. Then, it was conducted, respectively, in the aMCI and HC group to study the effects of genotype (MTHFR, APOE, and MTHFR × APOE). Age, gender, years of education, and TIV were set as covariates.

To further investigate the potential interaction effects between the MTHFR C677T and APOE genotypes, we conducted stratified analyses to detect the effects of diagnosis, MTHFR genotype, and diagnosis × MTHFR genotype among subjects with and without the APOE ε4 allele separately. Furthermore, we defined the brain regions with GM volume significantly affected by interaction effects as regions of interest and extracted their corresponding GM volume for further analysis.

Multiple comparison correction was performed using the cluster-level family wise error method, with statistical significance set to *p* < 0.05 corrected at the cluster level and *p* < 0.001 at the voxel-level.

## Results

### Participant Characteristics

The demographics, clinical, neuropsychological, and genotype characteristics are summarized in Table 1. No significant difference in gender (χ^2^ = 0.023, *p* = 0.881), age (*t* = 1.318, *p* = 0.191), and years of education (*Z* = −1.257, *p* = 0.209) was found between the aMCI group and the HC group. With regard to the vascular risk factors, the prevalence of smoking, alcohol, hypertension, diabetes, heart disease, or hyperlipidemia in both groups was comparable (*p* > 0.05). No significant difference in serum homocysteine (*Z* = −0.320, *p* = 0.749), folate (*t* = −0.001, *p* = 0.999), or vitamin B_12_ (*Z* = −0.293, *p* = 0.770) was observed between the aMCI and HC groups.

**TABLE 1:** Comparison of demographic, clinical, genotype, and neuropsychological characteristics between the amnestic mild cognitive impairment (aMCI) group and the healthy control (HC) group.

	aMCI (*n* = 60)	HC (*n* = 30)	χ ^2^*/t/Z*	*p* Value
Gender (male), n (%)	27 (45)	13 (43)	0.023^a^	0.881
Age, y	67.32 ± 9.44	64.67 ± 8.01	1.318^b^	0.191
Education, y	9 (7.25–11.75)	10 (8–13.25)	−1.257^c^	0.209
Smoking, n (%)	18 (30)	6 (20)	1.023^a^	0.312
Alcohol, n (%)	16 (27)	8 (27)	< 0.001^a^	1.000
Hypertension, n (%)	19 (32)	15 (50)	2.860^a^	0.091
Diabetes, n (%)	10 (17)	1 (3)	2.188^a^	0.139
Heart disease, n (%)	6 (10)	4 (13)	0.014^a^	0.906
Hyperlipidemia, n (%)	11 (18)	7 (23)	0.313^a^	0.576
Homocysteine, μmol/L	15.87 (13.42–20.6)	16.37 (12.68–19.55)	−0.320^c^	0.749
Folate, ng/mL	8.57 ± 3.66	8.57 ± 3.69	−0.001^b^	0.999
Vitamin B_12_, pg/mL	450.55 (336.78–566.33)	413.00 (348.10–542.28)	−0.293^c^	0.770
MMSE	24 (23–26)	28 (27.75–28)	−7.100^c^	< 0.001
MoCA	19.5 (17–23)	26 (24.75–27)	−6.532^c^	< 0.001
CAMCOG-C	76.5 (72–83)	90 (85–96)	−5.976^c^	< 0.001
Orientation	9 (7–10)	10 (9–10)	−4.160^c^	< 0.001
Language	25 (23–27)	27 (25.75–28)	−3.081^c^	0.002
Memory	13 (10.25–16)	20.5 (19–22)	−6.992^c^	< 0.001
Attention	6 (5–7)	7 (6–7)	−3.689^c^	< 0.001
Praxis	10 (8–11)	11.5 (10–12)	−3.040^c^	0.002
Calculation	2 (2–2)	2 (2–2)	−1.239^c^	0.215
Abstraction	6 (5–7)	6 (5.75–7)	−1.417^c^	0.156
Perception	6 (6–8)	8 (6.75–9)	−2.666^c^	0.008
GDS	4 (3–8)	4 (2–6)	−1.548^c^	0.122
ADL	20 (20–21)	20 (20–20)	−1.565^c^	0.118
TIV, cm^3^	1,338.19 ± 113.33	1,369.11 ± 131.29	−1.156^b^	0.251
**APOE, n (%)**				
ε4 carriers	22 (37)	8 (27)	0.900^a^	0.343
ε4 non-carriers	38 (63)	22 (73)		
**MTHFR, n (%)**				
T carriers	40 (67)	19 (63)	0.098^a^	0.754
CC	20 (33)	11 (37)		
**APOE ε 4carrier, n (%)**				
MTHFR T carriers	15 (68)	6 (75)	0.000^a^	1.000
MTHFR CC	7 (32)	2 (25)		
**APOE ε 4 non-carrier, n (%)**				
MTHFR T carriers	25 (66)	13 (59)	0.269^a^	0.604
MTHFR CC	13 (34)	9 (41)		

*aMCI, amnestic mild cognitive impairment; HC, healthy control; M, male; MMSE, Mini-Mental State Examination; CAMCOG-C, Cambridge Cognitive Examination-Chinese Version; MoCA, Montreal Cognitive Assessment; GDS, Geriatric Depression Scale; ADL, Activities of Daily Living scale; TIV, total intracranial volume.*

*^a^χ^2^ value of χ^2^ test.*

*^b^t value of two independent-samples t test.*

*^c^Z value of Mann–Whitney U test.*

Moreover, in terms of cognitive function, the aMCI group exhibited significantly lower MMSE score (*Z* = −7.100, *p* < 0.001), MoCA score (*Z* = −6.532, *p* < 0.001), total CAMCOG-C score (*Z* = −5.976, *p* < 0.001), and its subitem orientation score (*Z* = −4.160, *p* < 0.001), language score (*Z* = −3.081, *p* = 0.002), memory score (*Z* = −6.992, *p* < 0.001), attention score (Z = −3.689, *p* < 0.001), praxis score (*Z* = −3.040, *p* = 0.002), and perception score (*Z* = −2.666, *p* = 0.008) compared with the HC group, and no significant difference was observed in calculation score (*Z* = −1.239, *p* = 0.215), abstraction score (*Z* = −1.417, *p* = 0.156), GDS score (*Z* = −1.548, *p* = 0.122), and Activities of Daily Living score (*Z* = −1.565, *p* = 0.118) between the two groups. TIV in the two groups was similar (*t* = −1.156, *p* = 0.251).

The distributions of the APOE and MTHFR C677T genotypes in the aMCI group and HC group were comparable (χ^2^ = 0.900, *p* = 0.343; χ^2^ = 0.098, *p* = 0.754). In addition, no significant difference in the prevalence of MTHFR C677T variant was found in the aMCI group and HC group even when the subjects were stratified by APOE ε4 status (χ^2^ = 0.000, *p* = 1.000; χ^2^ = 0.269, *p* = 0.604).

### The Interactions of APOE-MTHFR on GM Volume

**FIGURE 1:**
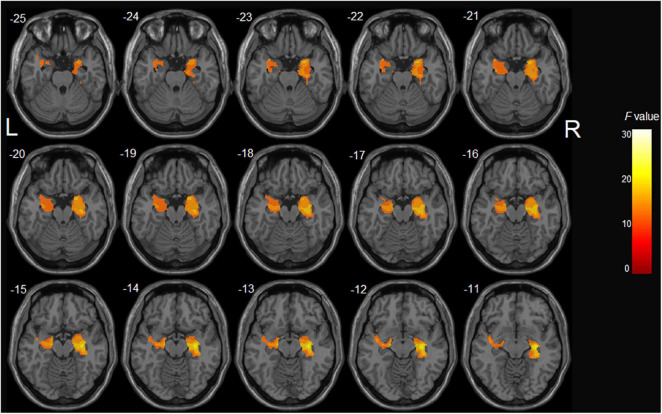
The main effect of diagnosis on gray matter volume in all participants: aMCI patients demonstrated significant gray matter atrophy in the bilateral hippocampus, right parahippocampal gyrus, and left superior temporal gyrus compared with healthy controls.

After controlling potential confounding factors, including age, gender, years of education, and TIV, the analysis involving all participants revealed a significant main effect of diagnosis (HC > aMCI) on the bilateral hippocampus, right parahippocampal gyrus, and left superior temporal gyrus (*p* < 0.05, corrected) (Table 2 and Figure 1). In contrast, the MTHFR C677T and APOE genotypes were not individually significantly associated with GM volume. Moreover, MTHFR × APOE and diagnosis × genotype interactions (diagnosis × MTHFR; diagnosis × APOE; diagnosis × MTHFR × APOE) had no significant effects on GM volume.

**TABLE 2:** Significant clusters showing the effects of aMCI and genotype on gray matter volume.

	Brain regions (AAL)	Cluster size (voxels)	Volumes, mm^3^	MNI coordinate			*F* value
				*X*	*Y*	*Z*	
**Diagnosis effect (aMCI < HC)**							
Cluster1	Left hippocampus	1,616	5,454.0	−18	−14	−14	22.05
	Left superior temporal gyrus			−41	−8	−11	17.39
Cluster2	Right hippocampus	3,202	7,204.5	32	−18	−9	30.55
	Right parahippocampal gyrus			23	3	−21	24.01
**APOE genotype effect in aMCI group (ε 4 carriers < ε 4 non-carriers)**							
	Right hippocampus	429	1,447.9	29	−36	−2	24.32
**Diagnosis x MTHFR C677T genotype in all subjects without APOEε 4 allele**							
	Right precuneus	81	273.4	17	−60	27	15.55

*AAL, anatomical automatic labeling; MNI, Montreal Neurological Institute; aMCI, amnestic mild cognitive impairment; HC, healthy control.*

**FIGURE 2:**
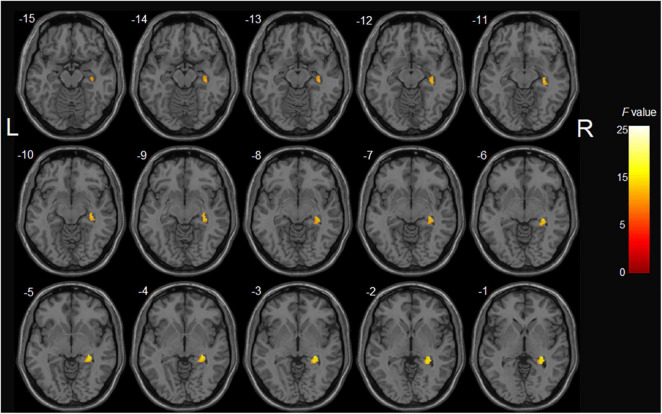
The main effect of APOE genotype on GMV in aMCI patients: among aMCI patients, APOE ε4 carriers demonstrated decreased gray matter volume of the right hippocampus compared with APOE ε4 non-carriers.

In the aMCI group, analysis showed significant GM volume reduction of the right hippocampus in APOE ε4 carriers compared with non-carriers (*p* < 0.001, uncorrected) (Table 2 and Figure 2). However, the main effect of MTHFR C677T genotype and epistatic interactions between MTHFR and APOE genotypes on the GM volume were not found. Furthermore, in the HC group, the MTHFR C677T and APOE genotypes and their epistatic interactions had no significant effects on GM volume.

**FIGURE 3:**
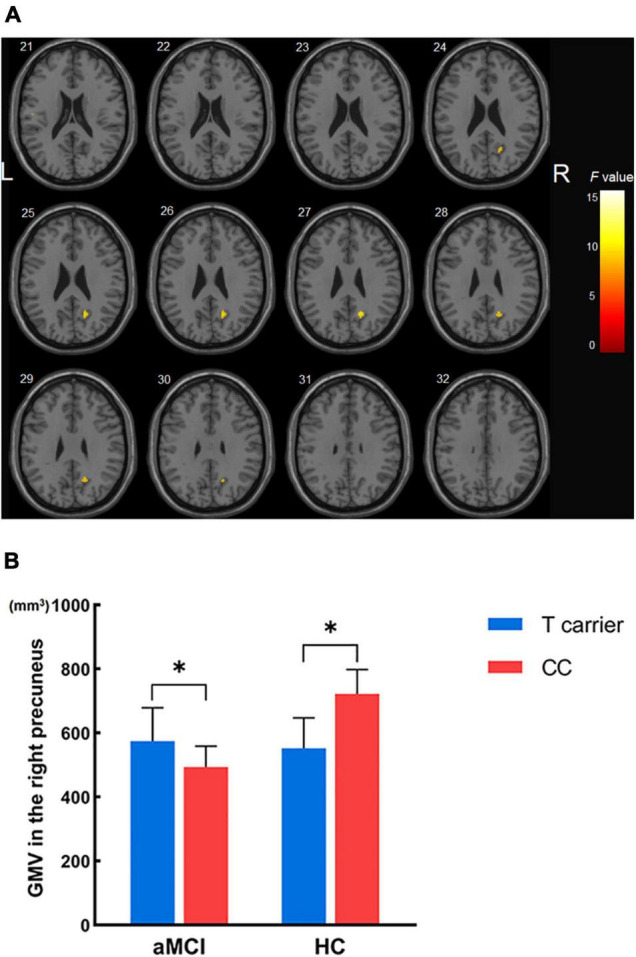
The interaction effects of diagnosis × MTHFR C677T genotype on gray matter volume in all subjects not carrying the APOE ε4 allele. **(A)** Significant interactions were found in the right precuneus. **(B)** Gray matter volume of the right precuneus across the four groups. *p < 0.05; GMV, gray matter volume.

The effects of diagnosis, MTHFR C677T, and diagnosis × MTHFR C677T interaction in different APOE genotype subgroups were further investigated. We initially found a significant main effect of diagnosis in the subgroup consisting of APOE ε4 carriers. aMCI patients with the APOE ε4 allele presented a significantly smaller right hippocampus volume than HCs that were APOE ε4 carriers (*p* < 0.05, corrected) (Table 3). However, no significant effects of the MTHFR C677T genotype itself and interaction of diagnosis × MTHFR genotype on the GM volume were found. Moreover, the analysis was conducted in a subgroup of APOE ε4 non-carriers, and a significant main effect of diagnosis was found (HC > aMCI, *p* < 0.001, uncorrected) (Table 3); however, the effect size was weaker than that found in the subgroup of APOE ε4 carriers. In addition, when we assessed the main effect of MTHFR C677T polymorphism on GM volume, no significant difference was found in any brain regions between the T carriers and CC homozygotes. Subsequently, we estimated the interaction effect on GM volume between diagnosis and MTHFR genotype and revealed a significant interaction on the right precuneus (*p* < 0.001, uncorrected) (Table 2 and Figure 3A).

**TABLE 3:** Diagnosis effect in all subjects with APOE ε4 allele and without APOE ε4 allele.

Brain regions (AAL)	Cluster size (voxels)	Volumes, mm^3^	MNI coordinate			*F* value
			*X*	*Y*	*Z*	
**Diagnosis effect in APOE ε 4 carriers (aMCI < HC)**						
Right hippocampus	704	2,376	35	−15	−11	32.53
**Diagnosis effect in APOE ε 4 non-carriers (aMCI < HC)**						
Right hippocampus	257	867.4	29	−17	−12	17.73
Right insula gyrus	131	442.1	39	20	−8	16.22
Left superior temporal gyrus	161	543.4	−45	6	−8	19.53

*AAL, anatomical automatic labeling; MNI, Montreal Neurological Institute; aMCI, amnestic mild cognitive impairment; HC, healthy control.*

Finally, we used simple effect tests to further explore the nature of the interaction effect. The right precuneus was defined as the region of interest and extracted its corresponding GM volume for further analysis as it was the brain region most significantly influenced by diagnosis × MTHFR genotype interaction. Importantly, our analysis revealed that in APOE ε4 non-carriers, a smaller right precuneus volume was found in HCs carrying the MTHFR T variant than in MTHFR CC homozygotes. In contrast with the HC group, a larger right precuneus volume was found in aMCI patients carrying the MTHFR T variant compared with MTHFR CC homozygotes (Figure 3B).

## Discussion

This is the first study to document the effects of epistatic interactions between the MTHFR C677T and APOE polymorphisms on GM atrophy in aMCI patients and healthy subjects to the best of our knowledge. We observed a significant main effect of APOE genotype on GM atrophy in the right hippocampus of aMCI patients, and the right precuneus could be influenced by the interaction between diagnosis and MTHFR C677T genotype in aMCI and HC subjects not carrying the APOE ε4 allele. These results provide structural imaging evidence of the effects of genetics in aMCI. Furthermore, the findings provide new insights into the complex roles of multiple genes in aMCI.

The bulk of research showed that GM abnormalities exist in patients with aMCI. Studies have demonstrated that aMCI patients exhibit more GM shrinkage of the hippocampus, parahippocampus gyrus, and temporal lobes (Whitwell et al., 2007). In line with these studies, the main effect of diagnosis in our study showed that aMCI patients experienced significant GM volume reduction of the hippocampus bilaterally, the right parahippocampal gyrus, and the left superior temporal gyrus, compared with HCs. Indeed, the hippocampus and parahippocampal gyrus belong to the medial temporal lobe (MTL) structure and are widely perceived as the initial brain regions affected in pathological studies of AD (Braak and Braak, 1991). GMV atrophy in the MTL is regarded as the neuroimaging hallmark of aMCI and has importantly been reported to be a neurostructural biomarker predicting the progression of aMCI to AD (Ferreira et al., 2011). Furthermore, functional neuroimaging studies also found that activation in the MTL and functional connectivity in subregions in aMCI patients were decreased compared with normal controls (Chen et al., 2016). Functionally, the above structures are generally considered to be the critical rendezvous points in a widespread cerebral network including the ventrolateral temporal lobe, medial and lateral parietal lobes, medial and lateral frontal lobes, and other cortical brain regions and are involved in the episodic memory encoding and retrieval (Dickerson and Eichenbaum, 2010). Notwithstanding that GM atrophy in aMCI has been extensively explored, little is currently known on the changes in GM volume caused by genetic factors.

Among multiple genetic factors associated with sporadic AD, the APOE gene is regarded as the most critical risk factor for AD. Most importantly, among the three isoform alleles (ε2, ε3, and ε4), APOE ε4 has been reported as the primary genetic risk factor (Liu et al., 2013) and associated with amyloid-β aggregation, synaptic plasticity, cholesterol homeostasis, neuroinflammation, and neurovascular functions (Bell et al., 2012). Several neuroimaging studies on AD and aMCI have shown smaller hippocampus volume and a faster rate of hippocampus atrophy in APOE ε4 carriers than non-carriers (Hashimoto et al., 2001; Schuff et al., 2009; Spampinato et al., 2011; Manning et al., 2014). Consistently, we found significant GM atrophy in the right hippocampus of APOE ε4 carriers compared with non-carriers in aMCI patients. It has been widely recognized that MTHFR C677T polymorphism is related to AD and brain structural alterations. In this regard, the MTHFR C677T variant has been associated with smaller regional brain volume in MCI patients (Rajagopalan et al., 2012). Furthermore, this variant has been associated with local brain atrophy in brain areas involved in intellectual and emotional functions, especially the medial orbitofrontal cortex (Roussotte et al., 2017). In addition, the MTHFR C677T variant can reportedly promote brain atrophy by increasing homocysteine levels (Rajagopalan et al., 2012; Roussotte et al., 2017). Interestingly, previous imaging studies showed that older adults with elevated homocysteine levels had more pronounced regional brain atrophy (Rajagopalan et al., 2011) and thinner cortical GM (Madsen et al., 2014). Besides, hyperhomocysteinemia has been associated with hippocampal atrophy in cognitively normal older adults (Williams et al., 2002; den Heijer et al., 2003) and more severe MTL atrophy in AD (Hooshmand et al., 2013).

Given that AD is a polygenic disorder, it is a matter of debate whether the interactions between MTHFR and APOE genes are related to the pathogenesis of AD (Tysoe et al., 1997; Chapman et al., 1998; Brunelli et al., 2001; Seripa et al., 2003; Wang et al., 2005; Keikhaee et al., 2006; Styczyńska et al., 2008; Bi et al., 2009; Mansouri et al., 2013). Extensive research has identified a correlation between MTHFR C677T polymorphism and the risk of AD, which, however, varied with the APOE ε4 carrier status (Bi et al., 2009; Zhang et al., 2010; Peng et al., 2015; Rai, 2017). This correlation has been documented to be stronger in APOE ε4 carriers (Bi et al., 2009; Roussotte et al., 2016). Interestingly, some studies found that the MTHFR C677T variant could influence susceptibility to AD in APOE ε4 non-carriers (Wang et al., 2005; Kim et al., 2008). One such meta-analysis based on 40 case–control studies revealed that MTHFR C677T polymorphism might contribute to the risk of AD, particularly in APOE ε4 carriers (Peng et al., 2015), whereas another meta-analysis, which included only well-designed case–control studies and strict diagnostic criteria, held that MTHFR C677T variant might also influence susceptibility to AD in APOE ε4 non-carriers (odds ratio = 1.27; 95% confidence interval = 0.97–2.02) (Zhang et al., 2010). In light of this, it would not be surprising that the correlation between MTHFR polymorphism and GM atrophy in aMCI patients was present only in APOE ε4 non-carriers in our current study. This finding can be explained by the following reasons. First, studies have shown that APOE ε4 is not only the most important genetic risk factor but also plays a dominant role in pathogenesis of sporadic AD, whereas MTHFR C677T is a relatively weak genetic risk factor for AD (Zuin et al., 2021). Our findings suggest that in APOE ε4 carriers, the MTHFR gene does not influence GM volume. Furthermore, we found the main effect of APOE gene on GM atrophy in aMCI patients; however, the main effect of MTHFR genotype on GM volume was not found in aMCI patients, nor did we find an interaction between MTHFR and diagnosis on GMV in participants with APOE ε4 carriers. Nevertheless, the results implicated the influence of the MTHFR genotype on the right precuneus volume in both aMCI patients and HCs not carrying the APOE ε4 allele. Second, the MTHFR CC genotype exerted a protective effect against AD, whereas the MTHFR 677T variant could accelerate AD progression by impairing homocysteine metabolism and promoting oxidative stress. In this regard, previous studies suggested that folate, a major regulatory factor for MTHFR activity and homocysteine levels, could quench oxidative damage (Shea and Rogers, 2002; Shea et al., 2002). Accordingly, abnormal metabolism of homocysteine and/or inappropriate folate intake may impair the oxidative stress response. The allele-specific antioxidant potential of APOE (ε2 > ε3 > ε4) has been substantiated in biological studies (Tamaoka et al., 2000; Colton et al., 2002), suggesting that the MTHFR CC genotype in APOE ε4 non-carriers leads to synergistic beneficial effects against oxidative stress. These findings suggest that individuals without the APOE ε4 allele are more susceptible to be influenced by MTHFR C677T polymorphism.

Herein, we found significant interaction effects between MTHFR C677T polymorphism and diagnosis on the right precuneus. The precuneus is a region of the posteromedial parietal lobe (Cavanna and Trimble, 2006) that plays an essential role in visuospatial imagery, episodic memory retrieval, self-processing, and consciousness and has various reciprocal connections with frontal, temporal, and parietal cortices (Cavanna and Trimble, 2006). Interestingly, it has been shown that changes in precuneus function may have pathophysiological relevance in aMCI development, with accumulating evidence indicating the presence of cortical thinning, metabolic alterations, and early amyloid deposition in the precuneus of aMCI patients (Bailly et al., 2015a,b; Coutinho et al., 2015; Csukly et al., 2016). In all APOE ε4 non-carriers of present study, a smaller right precuneus volume was found in healthy subjects who were MTHFR T carriers than healthy subjects that were MTHFR CC homozygotes. Intriguingly, opposite results were found in the aMCI group, with a larger right precuneus volume in MTHFR T carriers than MTHFR CC homozygotes. We hypothesized that this phenomenon might be related to adaptation and compensation during the process of aMCI development. Most importantly, our results corroborated that individuals without the APOE ε4 allele are more vulnerable to the effect of MTHFR C677T polymorphism, and structural alterations to the right precuneus could be influenced by the MTHFR C677T genotype in both aMCI patients and HCs who do not carry the APOE ε4 allele. MTHFR C677T polymorphism is reportedly the major genetic modifier associated with disorders of the folate cycle and homocysteine metabolism, which can be treated by supplementations of folate and vitamin B (Fohr et al., 2002; Du et al., 2018; Huang et al., 2018). There is still an ongoing debate on whether folate and/or vitamin B supplementation can improve cognition or slow the rate of brain atrophy in AD and/or MCI by lowing homocysteine levels (Durga, 2007; Kang et al., 2008; Smith et al., 2010; Ford and Almeida, 2012; Douaud et al., 2013; Clarke et al., 2014; Ma et al., 2019). Heterogeneity in findings of these studies may be influenced by the limitations during the selection of subjects, different lengths of intervention, and inconsistent inclusion criteria (Smith and Refsum, 2016). In addition, we speculate that the absence of APOE and MTHFR polymorphisms in these intervention trials may also be an important factor of poor intervention effects. Accordingly, future studies exploring the efficiency of homocysteine therapy tailored to different genetic backgrounds are needed to confirm our assumption.

There were several limitations in this study that should be considered. First, our sample size was limited, emphasizing the need to validate our findings in clinical studies with large sample sizes. Furthermore, given the limited number of aMCI patients enrolled, we could not further classify the aMCI patients into single-domain (aMCI-SD) or multidomain (aMCI-MD) (Petersen et al., 1999, 2009), which could impact the outcome of our study. Moreover, it should be noted that other genes associated with AD, including SCIMP, SLC2A4, CLU, and PICALM, were not taken into consideration in this study. Finally, we acknowledge that the observational imaging-genetic approach used in this cross-sectional study limits the causal inference.

## Conclusion

In summary, the current research provides preliminary evidence indicating that the interaction between diagnosis and MTHFR C677T polymorphism has a structural effect on the right precuneus in HCs and aMCI patients without the APOE ε4 allele. Confirmation of our findings in larger non-APOE ε4 cohorts is required. More studies should be conducted in the future, with study subjects stratified according to different genetic background, environmental exposure, or other risk factors, to clarify the possible role of MTHFR C677T polymorphism in the pathogenesis of AD and/or aMCI.

## Data Availability Statement

The original contributions presented in the study are included in the article/Supplementary Material, further inquiries can be directed to the corresponding author/s.

## Ethics Statement

The studies involving human participants were reviewed and approved by the Ethics Committees of the First Affiliated Hospital of Anhui Medical University (Reference no, Quick-PJ 2021-13-18). The participants provided their written informed consent to participate in this study.

## Author Contributions

MY performed the analysis and wrote the manuscript. MY and XZho conceived and designed the experiments. WY, KW, and WZha helped to collect neuropsychological data. CL, ML, and WZhu helped to perform MRI and collect MRI data. XZhu and ZS designed and supervised the study. All authors read and approved the final manuscript.

## Conflict of Interest

The authors declare that the research was conducted in the absence of any commercial or financial relationships that could be construed as a potential conflict of interest.

## Publisher’s Note

All claims expressed in this article are solely those of the authors and do not necessarily represent those of their affiliated organizations, or those of the publisher, the editors and the reviewers. Any product that may be evaluated in this article, or claim that may be made by its manufacturer, is not guaranteed or endorsed by the publisher.

## Footnotes

1
http://www.snpway.com/snpdecoder/

2
http://dbm.neuro.uni-jena.de/vbm8/

3
http://www.restfmri.net/forum/REST_V1.8
