# Supplementary material for: The Influence of MTHFR Polymorphism on Gray Matter Volume in Patients With Amnestic Mild Cognitive Impairment
Source: Front Neurosci. 2021 Nov 30;15:778123. doi: 10.3389/fnins.2021.778123 (PMC8670096; doi:10.3389/fnins.2021.778123)
Supplement: Supplementary file 1 [file Table_1.DOCX]

**Supplementary Materials**

**Table S1**. Sequencing primer of MTHFR rs1801133 and APOE rs429358, rs7412

| Primer name | Primer sequence | PCR product size(bp) |
| --- | --- | --- |
| rs429358_rs7412-F* | AGGAACAACTGACCCCGGTG | 326bp |
| rs429358_rs7412-R | CTGTTCCACCAGGGGCCC |  |
| rs1801133-F* | AGAGGACTCTCTCTGCCCAG | 295bp |
| rs1801133-R | CCCTCACCTGGATGGGAAAG |  |

* Sequencing SNP primers
